# Population aging and changing hospitalization risks in Germany: a decomposition of changes in inpatient cases, 2005–2021

**DOI:** 10.1186/s12889-026-27522-x

**Published:** 2026-04-30

**Authors:** Anna-Kathleen Piereth, Roland Rau, Marcus Ebeling

**Affiliations:** 1 Max Planck Institute for Demographic Research, Konrad-Zuse-Str. 1, Rostock, 18057 Germany; 2 Institute of Environmental Medicine, Unit of Epidemiology, Karolinska Institutet, Nobels Väg 13, Solna, Stockholm 171 77 Sweden; 3 Faculty of Economic and Social Sciences, University of Rostock, Ulmenstraße 69, Rostock, 18057 Germany; 4 Max Planck – University of Helsinki Center for Social Inequalities in Population Health (MaxHel Center), Rostock, Germany

**Keywords:** Inpatient cases, Population aging, Hospitalization risks, Total Hospitalization Rate, Decomposition, G-DRG system, Covid-19 pandemic, Germany, Demography

## Abstract

**Background:**

Inpatient care improves and maintains population health but requires a clear understanding of the drivers of inpatient care utilization. In Germany, inpatient cases increased from 16.5 to 19.4 million between 2005 and 2019 and dropped tremendously during the Covid-19 pandemic. We investigate how population aging and changing hospitalization risks in the German population contributed to the changes in inpatient case numbers remunerated according to the German Diagnosis-Related Groups (G-DRG) system between 2005 and 2021.

**Methods:**

The analysis is based on more than 280 million cases from the G-DRG Statistic and population exposures from the Human Mortality Database and covers all ages from 0 to 95+. We employ the Total Hospitalization Rate (THR) as the outcome, which measures total inpatient case numbers in relation to the changing population size. Using Kitagawa decomposition techniques, we quantify the impact of population aging and changes in age-specific hospitalization risks on THR changes before and during the Covid-19 pandemic, for the total population, women and men. To consider disease-specific influences on the age-specific hospitalization risks, we stratified the hospitalization risk effect by the disease category of the underlying primary diagnosis.

**Results:**

The THR experienced two distinct periods of change, increasing from 2005 to 2014 and decreasing afterwards, with a further drop related to the onset of the Covid-19 pandemic. Between 2005 and 2019, 64% of the increase in the THR can be attributed to the changing age structure. Population aging led to an increase in inpatient cases throughout the observation period, whereas the effect of changing age-specific hospitalization risks reversed from an increase in cases between 2005 and 2014 to a decrease in cases between 2014 and 2021. This trend shift has offset the effects of population aging since 2014 and was excessively strong during the Covid-19 pandemic. The decline in age-specific hospitalization risks across many disease categories underlines that no specific health conditions are responsible for the trend change in hospitalization risks.

**Conclusions:**

Our findings demonstrate the importance of population aging for inpatient case numbers. Future hospital planning needs to consider the central role of population aging in shaping inpatient care needs.

**Supplementary Information:**

The online version contains supplementary material available at 10.1186/s12889-026-27522-x.

## Introduction

Inpatient care improves and maintains population health as a fundamental pillar of the healthcare system. For hospital care planning, understanding the drivers of inpatient case numbers is crucial. With demographic change and especially population aging, insights into the determinants of changing case numbers are becoming increasingly important [1, 2]. In Germany, inpatient cases increased from 16.5 to 19.4 million (+ 17%) between 2005 and 2019 and dropped dramatically during the Covid-19 pandemic [3]. This rise has been considered excessive given that the total German population increased by only 1%, from 82.4 to 83.2 million, during the same period [4]. The mechanisms underlying the increase in inpatient cases have been frequently assessed and are still controversially debated [5–13], particularly with regard to population aging [14]. This study adds a demographic perspective on the dynamics of inpatient cases over the last two decades. It quantifies the impact of changes in the age structure of the German population and changes in age-specific hospitalization risks.

Previous research has distinguished the drivers of changes in inpatient cases into demand-side determinants (i.e., population characteristics such as age structure and morbidity prevalence) and supply-side determinants (e.g., hospital bed capacity, availability of medical-technical innovations, incentives of the hospital reimbursement system). Earlier studies concluded that demand-side determinants together explained only about 20% of the increase in inpatient cases [12, 13]. It was thus suggested that supply-side determinants were primarily responsible, especially the economic incentives of the German Diagnosis-Related Groups (G-DRG) system for hospitals to treat more patients, following its introduction in 2004 [15]. Among the demand-side factors, changes in morbidity and changes in the size of the surviving population were identified as the most and second most important factors, respectively [12, 13].

However, prior research has left two significant gaps. *First*, changes in the absolute number of inpatient cases were used as the outcome measure. This outcome is highly problematic because it does not account for the change in size and structure of the underlying population [16, 17]. To understand whether rising cases result from (I) population aging or (II) an actual increase in the risk of hospitalization, inpatient case numbers must be linked to the population. This can be achieved by using hospitalization rates. For this, inpatient cases are related to the population at risk of hospitalization. For example, a hospitalization rate of 0.8 means that 80 inpatient cases occurred for every 100 person-years of exposure. *Second*, previous research has examined changes in inpatient cases over relatively short periods. Because demographic processes typically evolve slowly, the impact of population aging should be studied over longer observation periods to be fully understood.

We argue that population aging is a key driver of inpatient case numbers. As the population ages, more individuals are reaching advanced ages where hospitalizations are more likely [18, 19]. In Germany, the strongly populated cohorts 1948–1958 and the “baby boomers” (1959–1968) shifted to advanced ages between 2005 and 2019, with all these populous cohorts having exceeded at least age 50 by 2019. If everything else stays the same, these dynamics alone would lead to increasing inpatient case numbers. At the same time, changes in the age-specific hospitalization risks may affect inpatient cases. Changing hospitalization risks reflect the influence of various demand- and supply-side factors. For instance, a rising prevalence of morbidity or an improved availability of inpatient care could lead to higher age-specific hospitalization risks, whereas improvements in population health may lead to decreasing risks. An increase in age-specific hospitalization risks would mean that a life year lived at a certain age in Germany involves, on average, more hospital stays, while the opposite is true for decreasing risks.

The key contribution of the article is to quantitatively disentangle how much of the dynamics of inpatient case numbers can be attributed to a) population aging and b) changes in the age-specific hospitalization risks. Therefore, we employ an outcome that measures the total number of inpatient cases in relation to the population size, which we call the “Total Hospitalization Rate” (THR). This metric is standard in international hospital statistics, though it is referred to by different names, such as “hospital discharge rate” or “discharges per 100,000”. Our focus is on inpatient cases at all ages during the years 2005 to 2021 that were remunerated according to the German Diagnosis-Related Groups (G-DRG) system. To our knowledge, this is the first study to decompose changes in inpatient case numbers in Germany over a 17-year period.

## Methods

### Data

Information on inpatient cases was derived from the German Diagnosis-Related Groups (G-DRG) Statistic for the years 2005 to 2021 [20, 21]. The G-DRG Statistic is an annual survey of all hospital cases accounted for by the G-DRG system, which has been mandatory for general hospitals since 2004. The G-DRG Statistic can be regarded as an almost complete enumeration of acute care in German hospitals. It excludes only hospital care services charged by psychiatric and psychosomatic institutions in accordance with § 17 d (1) KHG (Act on the Economic Security of Hospitals and the Regulation of Hospital Nursing Rates) [22] which is a minor fraction (about 6%) of the total hospital care utilization. The data of the G-DRG Statistic is collected by the Institute for the Hospital Remuneration System (InEK) for accounting purposes, which submits a legally defined variable set to the Federal Statistical Office. Starting from the survey year 2005, access to the microdata of the G-DRG Statistic can be requested via the Research Data Centers of the Federal Statistical Office and the Statistical Offices of the Federal States (further information can be found on the website: https://www.forschungsdatenzentrum.de/en/health/drg). We grouped inpatient cases by sex and single years of age between 0 and 94, and the open-ended age group of 95 +. Cases were also stratified by primary diagnosis according to the main chapter of the 10th revision of the International Classification of Diseases, German Modification (ICD-10-GM). Person-years by age and sex were extracted from the Human Mortality Database [23, 24].

### Inclusion and exclusion criteria

We only included patients who stayed in the hospital overnight (inpatients). Thus, “day cases” and cases which had been transferred to another hospital were excluded from the analysis. We also restricted the analysis to cases with residence in Germany and with valid information on the patients’ demographics and on the hospital stay. Cases with missing information on age, sex, cause of discharge, and those with an error DRG code were excluded. Moreover, cells with less than three observations were blocked by the data provider due to confidentiality rules [25]. However, these omitted cases made up only a negligible share of the original sample (0.0003%). A total of 280,654,809 inpatient cases (93.7% of the original sample) could be included in the analysis. Notably, the proportion of cases in each exclusion criterion varied only negligibly by survey year, as did the proportion of the original sample included (see Additional file 1: Table S1).

### Analytical strategy

*In the first* *step* of the analysis, we compared the aging of inpatients and the total German population over time by their respective mean ages. *In the second step*, we calculated the "Total Hospitalization Rate" (THR) to measure inpatient cases in relation to the population size. The THR is calculated by dividing the total number of inpatient cases (H) by the total person-years lived (N) in a given year:

1 $$THR=\frac{H}{N}$$

The THR thus may be interpreted as the average number of hospitalizations that an average person-year lived involves in a certain year. The THR is expressed as cases per 100,000 inhabitants, in accordance with international standards for the reporting of any inpatient discharge rates, which are a key metric of hospital utilization and follow the same definition as the THR [26]. In our study, the total number of inpatient cases (H) is equivalent to the total number of discharged patients because death during hospitalization is considered a cause of discharge in the G-DRG Statistic. *Subsequently*, we used the THR twofold:

I) To investigate the time trend without the impact of the changing age structure, the THR was age-standardized using the German population of 2011 as the standard [27]:

2 $$ASTHR={\sum }_{i=0}^{95+}{h}_{i}\cdot {c}_{i}^{s}$$

In formula (2), “${h}_{i}$“ represents the age-specific hospitalization risk at age *i*. This is calculated by dividing the number of hospitalizations occurring at age *i* by the number of person-years lived at age *i* in a given year. The term ‘age-specific hospitalization risks’ is used throughout to denote ‘age-specific hospitalization rates’, for ease of understanding. “${c}_{i}^{s}$“ denotes the proportion of the standard population falling in age *i*.

II) We decomposed changes in the THR over time using the method proposed by Kitagawa [28]:

3 $$\begin{aligned}\Delta &={THR}^{{t}_{2}}-{THR}^{{t}_{1}}={\sum\limits}_{i=0}^{95+}({c}_{i}^{{t}_{2}}-{c}_{i}^{{t}_{1}})\left(\frac{{h}_{i}^{{t}_{2}}+{h}_{i}^{{t}_{1}}}{2}\right)\\&+{\sum\limits}_{i=0}^{95+}({h}_{i}^{{t}_{2}}-{h}_{i}^{{t}_{1}})\left(\frac{{c}_{i}^{{t}_{2}}+{c}_{i}^{{t}_{1}}}{2}\right)\end{aligned}$$

The Kitagawa decomposition breaks down the difference in THR ($\Delta$) between two time points (${t}_{1}$ and ${t}_{2}$) in a given population into two components. The first summand represents the effect of the changing age structure between time points ${t}_{1}$ and ${t}_{2}$, while the second summand denotes the effect of changing age-specific hospitalization risks between those time points. The total THR change (${THR}^{{t}_{2}}-{THR}^{{t}_{1}}$) is simply the sum of the two contributions, which are based on the proportion of the population at age *i* at time points ${t}_{1}$ and ${t}_{2}$ (${c}_{i}^{{t}_{1}}, {c}_{i}^{{t}_{2}}$) and the age *i*-specific hospitalization risks of the population at time points ${t}_{1}$ and ${t}_{2}$ (${h}_{i}^{{t}_{1}}, {h}_{i}^{{t}_{2}}$).

An example: If the THR increases by 1,000 cases per 100,000 inhabitants between years one and two, this increase may be partially attributed to population aging, which could contribute 700 cases per 100,000 inhabitants and thus account for 70% of the THR change. The remaining 30% of the THR change could be attributed to rising age-specific hospitalization risks, which would make up an increase of 300 cases per 100,000 inhabitants. The presented decomposition allows us to quantify both effects.

We chose the Kitagawa decomposition because it allows the use of a rate as the outcome measure, which sets hospitalizations in relation to the underlying population size. Therefore, changes in the THR reflect changes in the intensity of inpatient care utilization. The THR implicitly incorporates changes in population size, which were found to play only a marginal role in changing the absolute number of inpatient cases in Germany [1, 11]. Moreover, as a summative decomposition with only two components, it is advantageous in terms of its parsimony. Compared to other decomposition methods used in the context of hospitalization [1, 8, 11], it does not introduce extra complexity due to additional factors without a substantial contribution to the outcome difference. The Kitagawa decomposition allows the distinct components to be represented in absolute and relative units. Since it does not require any residual category or interaction terms, the two contributions always add up to the outcome difference, facilitating interpretation.

To consider disease-specific influences on the age-specific hospitalization risks [29, 30], we additionally stratified the contributions of the changing hospitalization risks by the disease category of the underlying primary diagnosis:

4 $${\sum\limits}_{i=0}^{95+}\left({h}_{i}^{{t}_{2}}-{h}_{i}^{{t}_{1}}\right)\left(\frac{{c}_{i}^{{t}_{2}}+{c}_{i}^{{t}_{1}}}{2}\right)= {\sum\limits}_{j=A}^{Z}{\sum\limits}_{i=0}^{95+}\left[({h}_{i,j}^{{t}_{2}}-{h}_{i,j}^{{t}_{1}})\left(\frac{{c}_{i}^{{t}_{2}}+{c}_{i}^{{t}_{1}}}{2}\right)\right]$$

“${h}_{i,j}^{t}$“ represents the risk of being hospitalized at age *i* for a primary diagnosis of the ICD main chapter *j* at timepoint *t*. The sum of all age- and disease-specific hospitalization risks across ICD main chapters (A to Z) equals the total age-specific hospitalization risk (${\sum\limits}_{j=A}^{Z}{h}_{i,j}^{t}={ h}_{i}^{t}$). The results after stratification are presented for the five most prevalent disease categories (diseases of the circulatory system (ICD-10-GM: I00-I99), malignant neoplasms (C00-C97), diseases of the digestive system (K00-K93), diseases of the musculoskeletal system and connective tissue (M00-M99), injuries (S00-S99)), pregnancy, childbirth, and the puerperium (O00-O99), and all other. All analyses were conducted for the total population and separately for women and men. The decompositions were additionally performed for the time intervals 2005–2019, 2005–2014, 2014–2019, and 2019–2021 to investigate time trends. The analyses were performed using R (version 4.4.0) [31].

## Results

### Aging of inpatients and the general population

**Fig. 1:**
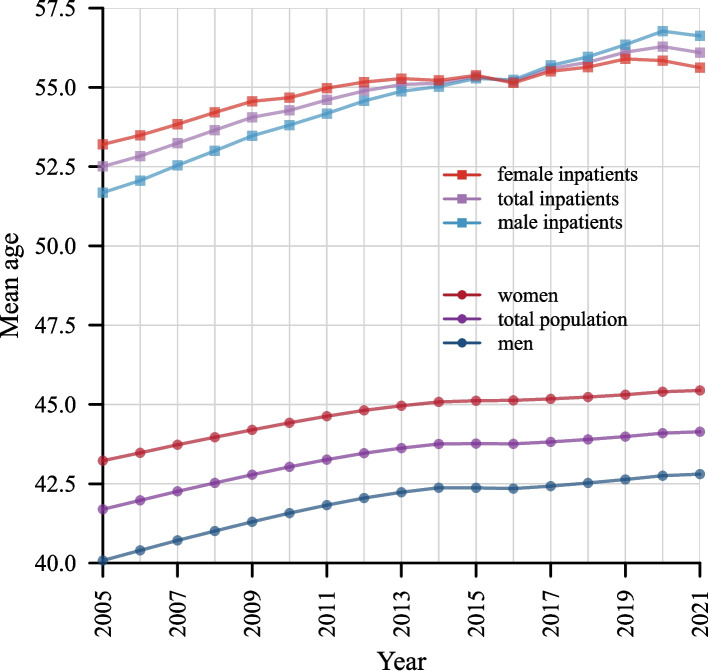
Mean age of inpatients and the general population, by sex (2005–2021)

Figure 1 illustrates the development of the mean age of inpatients and the general population from 2005 to 2021. Inpatients are on average not only considerably older, but also age more rapidly than the general population. Specifically, the difference in mean age was 10.8 years in 2005, widening to 12.0 years in 2021. This was due to a faster increase in the mean age of inpatients, which rose from 52.5 to 56.1 years, compared to an increase from 41.7 to 44.1 years in the total population. While the mean age of women was consistently higher than that of men, the mean ages of male and female inpatients showed a crossover, with the mean age of male inpatients exceeding that of female inpatients from 2016 onwards.

### Time trends in the Total Hospitalization Rate

**Fig. 2:**
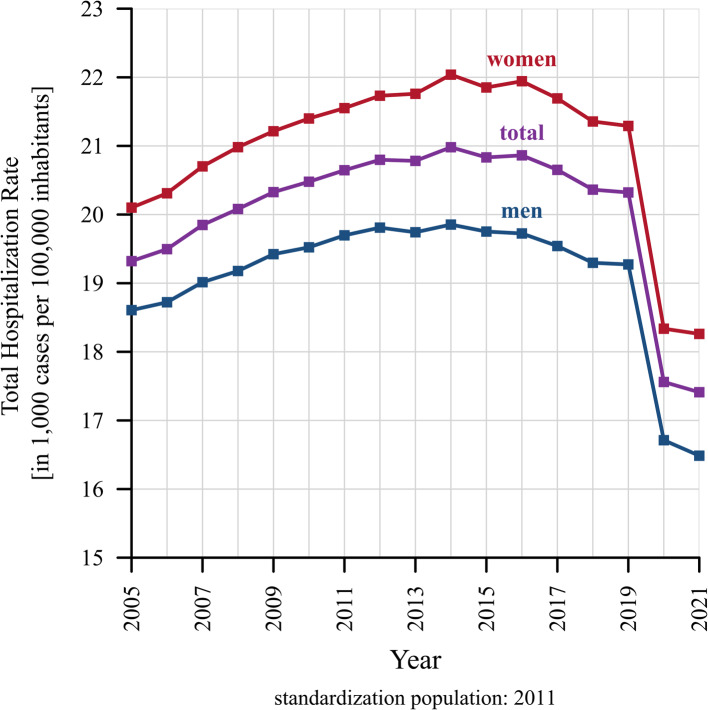
Age-standardized Total Hospitalization Rate, by sex (2005–2021) – The Total Hospitalization Rate (THR) measures the total number of inpatient cases divided by the total person-years at risk of hospitalization for each year, expressed as per 100,000

Figure 2 displays the age-standardized Total Hospitalization Rate in 1,000 cases per 100,000 inhabitants, for the total population as well as for women and men from 2005 to 2021. The age-standardized THR of the total population increased steadily from about 19,300 cases in 2005 to a maximum of about 21,000 cases in 2014. Between 2014 and 2016, the age-standardized THR stagnated at around this level, followed by a downward trend that resulted in about 20,300 cases in 2019. This decline accelerated with the onset of the Covid-19 pandemic, reaching a minimum of about 17,400 cases in 2021 which is below the level of 2005. The sex-specific age-standardized THR showed a similar pattern, whereby the age-standardized THR in women was continuously about 2,000 cases higher than in men. The year 2014 could be identified as a turning point between a period of increase and a period of decrease in the inpatient cases in relation to the population size, while 2019 marked the last year before the drop in cases during the Covid-19 pandemic.

### Decomposition of changes in the Total Hospitalization Rate

**Fig. 3:**
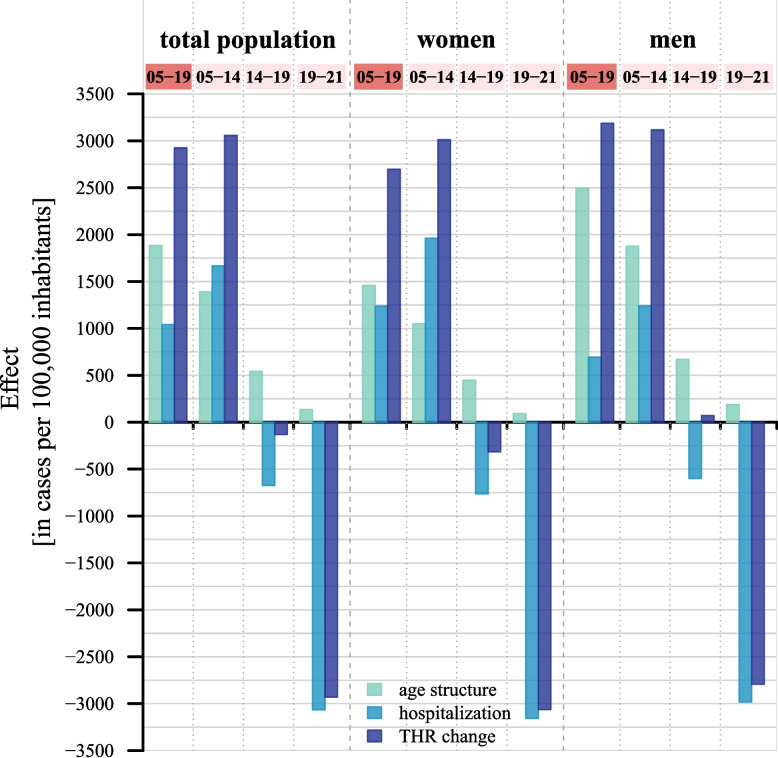
Kitagawa decomposition of changes in the Total Hospitalization Rate into age structure effects and hospitalization risk effects – time periods 2005–2019 (highlighted in red), 2005–2014, 2014–2019, and 2019–2021 (all highlighted in light rose), total population and by sex – exact absolute and relative contributions are provided in Table 1

The effects of changes in age structure and of changes in hospitalization risks on the change in the THR in cases per 100,000 inhabitants are shown in Fig. 3 and Table 1. The three bars on the upper left display the results for the total population, comparing the changes between 2005 and 2019. The THR increased by 2,926 cases per 100,000 inhabitants from 2005 to 2019 (dark blue bar), which corresponds to an increase of the THR by 15.9% over the considered period. The change in age structure contributed to an increase of 1,884 cases (light blue bar). This accounts for 64% of the total growth in the THR. The change in hospitalizations risks had a smaller effect with a contribution of 1,041 cases (see bar in blue). Population aging also emerged as the main driver of the THR growth in the sex-specific decompositions, with a higher contribution in men (2,494 cases) compared to women (1,459 cases). For women and men, rising age-specific hospitalization risks also contributed to the THR increase between 2005 and 2019. The THR would have increased by only 6.4% or rather 1,238 cases in women and by 4.0% or rather 694 cases in men, if only hospitalization risks had changed.

**Table 1:** Kitagawa decomposition of changes in the Total Hospitalization Rate into age structure effects and hospitalization risk effects - the absolute and relative contributions of the age structure effect and hospitalization risk effect are shown for the time periods 2005–2019, 2005–2014, 2014–2019, and 2019–2021 for the total population and by sex

**Period**		**Total population**		**Women**		**Men**	
		(cases per 100,000 inhabitants)	(%)	(cases per 100,000 inhabitants)	(%)	(cases per 100,000 inhabitants)	(%)
2005–2019	THR 2019	21,314		22,110		20,496	
	THR 2005	18,388		19,413		17,309	
	THR change	2,926	15.91	2,697	13.89	3,187	18.41
	Age structure effect	1,884	10.25	1,459	7.52	2,494	14.41
	Hospitalization risk effect	1,041	5.66	1,238	6.38	694	4.01
2005–2014	THR 2014	21,445		22,425		20,426	
	THR 2005	18,388		19,413		17,309	
	THR change	3,057	16.62	3,012	15.52	3,117	18.01
	Age structure effect	1,391	7.56	1,050	5.41	1,877	10.84
	Hospitalization risk effect	1,666	9.06	1,962	10.11	1,241	7.17
2014–2019	THR 2019	21,314		22,110		20,496	
	THR 2014	21,445		22,425		20,426	
	THR change	−131	−0.61	−315	−1.40	70	0.34
	Age structure effect	543	2.53	448	2.00	670	3.28
	Hospitalization risk effect	−674	−3.14	−763	−3.40	−599	−2.93
2019–2021	THR 2021	18,383		19,045		17,704	
	THR 2019	21,314		22,110		20,496	
	THR change	−2,931	−13.75	−3,065	−13.86	−2,792	−13.62
	Age structure effect	136	0.64	93	0.42	189	0.92
	Hospitalization risk effect	−3,066	−14.38	−3,158	−14.28	−2,982	−14.55

Note: The relative contributions are calculated by dividing the THR change, age structure effect, and hospitalization risk effect by the Total Hospitalization Rate observed at the beginning of the respective period

The subperiod-specific decompositions show that the THR change shifted from an increase between 2005 and 2014 to a decrease from 2014 onwards. This was due to a trend reversal in the direction of the hospitalization risk effects, which were positive between 2005 and 2014 and negative from 2014 onwards. In contrast, population aging had a consistently positive effect on the THR throughout the subperiods. For example, in the total population, there were contributions of 1,391 cases between 2005 and 2014, and of 543 cases between 2014 and 2019. The major drop in the THR in the three-year period 2019–2021 was comparable in size to the increase during the ten-year period 2005–2014. However, it was almost fully driven by the stark decline in hospitalization risks associated with the onset of the Covid-19 pandemic. This hospitalization risk effect of −3,066 cases resulted from universally negative contributions of all ages: 0–19 (−326 cases); 20–39 (−356 cases); 40–59 (−634 cases); 60–79 (−1,133 cases); and 80 and above (−618 cases). Overall, the pattern of the THR changes in the total population was evident in the sex-specific analysis, with slightly varying magnitudes of the age structure and hospitalization effects.

**Fig. 4:**
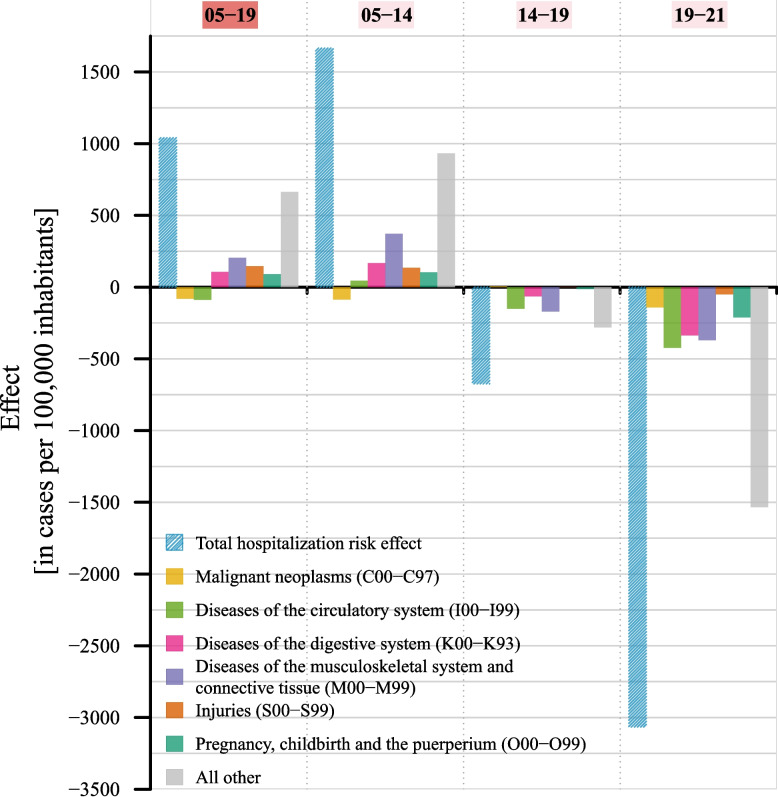
Stratification of the hospitalization risk effect by disease category, total population – the five most prevalent disease categories are presented for the time periods 2005–2019 (highlighted in red), 2005–2014, 2014–2019, and 2019–2021 (all highlighted in light rose): diseases of the circulatory system (ICD-10-GM: I00-I99), malignant neoplasms (C00-C97), diseases of the digestive system (K00-K93), diseases of the musculoskeletal system and connective tissue (M00-M99), injuries (S00-S99), as well as the disease category pregnancy, childbirth, and the puerperium (O00-O99), and all other – sex-specific results are illustrated in Additional file 2: Figure S1–S2 – exact absolute and relative contributions are provided in Additional file 3: Table S2 – *ICD-10-GM* 10th revision of the International Classification of Diseases, German Modification

Figure 4 presents the hospitalization risk effects from Fig. 3 (blue bars) for the total population, broken down by the five most prevalent disease categories, plus pregnancy, childbirth, and the puerperium, and all other. From 2005 to 2019, the positive hospitalization effect was driven by an increase in age-specific hospitalization risks for most disease groups. However, diseases of the circulatory system and malignant neoplasms showed negative contributions, preventing the THR from rising even further. In the subperiods 2005 to 2014 and 2014 to 2019, the changes in hospitalization risks for malignant neoplasms did also not align with the direction of the total hospitalization effect, showing a negative contribution and an effect close to zero, respectively. Of the presented disease categories, decreases in the hospitalization risks for musculoskeletal diseases accounted for the largest share of the total hospitalization effect in the period 2005 to 2019 as well as in the subperiods 2005 to 2014 and 2014 to 2019. The substantial decline in the hospitalization effect between 2019 and 2021 was driven by universally decreasing hospitalization risks across all disease categories (see Additional file 4: Table S3).

## Discussion

Germany experienced a rise in inpatient cases from 16.5 million to 19.4 million (+ 17%) between 2005 and 2019, followed by a steep decline during the Covid-19 pandemic. The mechanisms underlying these changes have remained unclear, also because they could not simply be traced to changes in the size of the German population, which increased by only 1%, from 82.4 to 83.2 million, over the same period. This study analyzed whether the dynamics of case numbers are due to: (I) more people reaching advanced ages where hospitalizations are more likely (i.e., population aging), or (II) changing hospitalization risks as a consequence of altering demand-side factors (other than population aging) and supply-side factors. Unlike previous analyses focusing on absolute case numbers only, we employed the Total Hospitalization Rate (THR) as the outcome measure. It relates the total number of inpatient cases to the total population size. The THR enabled us to assess whether an average life year in Germany has become more or less likely to involve an inpatient stay, and to disentangle the demographic and non-demographic drivers behind this development.

Our study revealed four main findings. *First,* inpatients are on average not only considerably older but also age more rapidly than the total population. *Second*, the THR evolved over time with distinct periods of change. It increased steadily from 2005 to 2014, decreased in subsequent years, and dropped even further with the onset of the Covid-19 pandemic. Consequently, an average life year in Germany in 2021 involved fewer hospital stays than in 2005. Notably, the observed THR pattern resulted primarily from the dynamics in inpatient case numbers (see Additional file 5: Figure S3), while changes in the size of the German population did not substantially vary throughout the study period [32]. *Third*, population aging emerged as a main driver of the inpatient case increase. We found that population aging accounted for 64% of the THR increase in the total population between 2005 and 2019. This challenges previous studies that undermined the role of population aging as a determinant of inpatient case numbers [12, 13]. *Fourth*, there was a trend change in the age-specific hospitalization risks, which were rising between 2005 and 2014 and declining from 2014 onwards. This trend break occurred across all ages and the most disease categories requiring hospitalization.

Our findings demonstrate that the age structure of the German population sets the stage for inpatient case dynamics. Population aging made a substantial and consistently positive contribution to the THR development throughout 2005 to 2021. This is because hospitalizations concentrate in the second half of the lifespan, when the incidence and prevalence of aging-related diseases also increase [18]. As life expectancy rises, more individuals reach these high-risk ages, which, all else being equal, drives inpatient cases upward. Men exhibited a stronger age structure effect than women, which is consistent with their greater gains in life expectancy between 2005 and 2019 (2.4 years vs. 1.5 years) [33]. Considering that men have a higher hospitalization risk beyond age 50 compared to women [19], the larger increase in male life expectancy may have resulted in the mean age of male inpatients rising faster than that of female inpatients, and surpassing it since 2016. Since the pace of population aging varies widely across German regions [34], the demographic impact may be even stronger in areas with faster population aging, like rural regions. Nationally, the aging of large birth cohorts, such as the “baby boomers”, combined with improving survival at older ages, will likely amplify the demographic effects on inpatient cases [35].

Therefore, the results question the established practices of hospital care planning in Germany. To calculate the required number of hospital beds, the federal states use the “Hill-Burton Formula”. It is based on the parameters population size, number of cases, length of stay, and bed utilization rate [36, 37]. However, the formula does not account for the current and future age structure of the population living in a specific area. As population aging plays a central role for hospital care utilization, demographic parameters should become a standard quantity in hospital planning frameworks. To provide efficient inpatient care, the implementation of the ongoing hospital reform [38, 39] should build on profound demographic evidence regarding the future number and age composition of hospital patients.

To our knowledge, this is the first study to document the decrease in age-specific hospitalization risks since 2014. This phenomenon might be understood in the context of the G-DRG system introduction in 2004. Hospitals may have adapted their practices to the case-increasing incentives of the new reimbursement system during the first years after its introduction. Supporting evidence comes from the stagnation of inpatient cases observed before the DRG reform (see Additional file 5: Fig. S3). By 2014, these adjustment processes and the corresponding increase in age-specific hospitalization risks might have largely been concluded. The following decline in hospitalization intensities may indicate improving population health in Germany, alongside the effects of other demand- and supply-side factors [40, 41]. Such improvements in population health may have been supported by medical progress in various areas. For example, the 2015 Prevention Act ("Präventionsgesetz") aimed to strengthen health promotion and disease prevention, as well as develop a cross-sector prevention strategy at the national level [42]. Other efforts include disease-specific action plans that promote the development and application of evidence-based guidelines in everyday clinical practice, such as the national cancer plan ("Nationaler Krebsplan") initiated in 2008 [43]. Additionally, there has been an increased utilization of new medical technologies in care-providing institutions, such as hospitals [44]. However, the combined effect of these achievements is expected to have a successive rather than sudden impact on hospitalization risks at the population level. Therefore, it may not fully explain a trend reversal in age-specific hospitalization risks, but it paves the way for a continuous decline in these risks over time. Finally, the sharp decrease in age-specific hospitalization risks since 2020 may be more due to supply-side factors than to declining healthcare needs, which tend to evolve gradually rather than abruptly. In 2024, hospital cases were still 10% below the pre-pandemic level. However, the evidence remains scarce in explaining the absence of a rebound in case numbers after the pandemic and its effects on the utilization of other healthcare system sectors and population health [45, 46]. Therefore, further research is needed to critically assess the long-lasting downward trend in inpatient cases that began already six years before the start of the Covid-19 pandemic.

Although our analysis did not determine the reasons for the declining hospitalization risks, it allowed us to exclude some explanations: The decrease cannot be attributed to changes in the proportion of day cases, which remained remarkably stable at around only 2.8% throughout the study period (see Additional file 1: Table S1). Therefore, a redistribution of inpatient services (especially 'ambulatory-sensitive case conditions') to day cases or the outpatient sector cannot be considered as a plausible reason. This is further emphasized by the slow progress in shifting inpatient to outpatient care in Germany, as described elsewhere [42, 47, 48]. For example, the number of outpatient surgeries both in the inpatient and outpatient sector did hardly change between 2006 and 2022 [47]. To the best of our knowledge, there were also no policy changes related to healthcare system structures that could have caused a trend reversal towards decreasing hospitalization risks. The only major reform potentially affecting the hospital sector was the Hospital Structure Act (“Krankenhausstrukturgesetz” (KHSG)) passed in 2015. It aimed at introducing a quality-based hospital financing and the fulfillment of quality requirements by hospitals as a criterion for their inclusion and retention in the state hospital plans [42]. However, the outcomes of this law were evaluated as ineffective [48]. Changes in time to death cannot serve as an explanation either: If the number of individuals in their last year of life had been the driving force behind the THR development (following the so-called "red herring" hypothesis [12]), the THR would have risen steadily between 2005 and 2021, as the absolute number of deaths in Germany increased continuously during that period [49]. However, the discontinuous THR pattern points to other driving factors, as discussed above. The decline in age-specific hospitalization risks across many disease categories underlines that no specific health conditions are responsible for the trend change in the THR.

### Strengths and limitations

This study has several notable strengths. Our investigation spans a 17-year period, capturing both the increase (2005–2014) and subsequent stagnation and decline (2014–2021) in the THR within a single analysis. This has never been done in previous research. Additionally, our use of hospitalization risks rather than absolute numbers allows for a more precise linkage of population dynamics to changes in hospitalizations compared to analyses using counts only. However, several limitations should be acknowledged. Since our analysis rests on the G-DRG Statistic, which does not represent the entire inpatient care sector (for example, it does not fully cover psychiatric diagnoses), our conclusions are limited to inpatient case numbers remunerated according to the G-DRG system. Furthermore, our analysis could only begin with the start of the G-DRG Statistic in 2005 preventing examination of earlier trends that could offer important context for the observed patterns. Data for 2022 and 2023 were also not available to us, which limits our findings mostly to pre-pandemic years. Methodologically, our decomposition assigns all influences beyond aging to the hospitalization risk effect. This prevents us from disentangling morbidity-driven demand changes from supply-side factors. It also precludes us from drawing conclusions about the relationship between hospitalization and mortality dynamics. Hospitalizations may not only be considered as a driver of increasing life expectancy but also as a consequence of changing cause-specific mortality. Furthermore, this relationship may vary across countries with different cause-of-death profiles and healthcare system types. Therefore, further research is needed to simultaneously address the interplay between age-specific hospitalization risks, health outcomes, mortality improvements, and demographic shifts.

## Conclusions

Our study offered a demographic perspective on the dynamics of inpatient cases in Germany over the last two decades. The results revealed that population aging is a key driver with a consistently increasing effect on case numbers. However, the impact of age-specific hospitalization risks was period-specific and has had a decreasing effect on inpatient cases since 2014, reducing the impact of population aging. Declining hospitalization risks have the potential to mitigate future challenges in providing adequate inpatient care to an aging population. Underlying reasons should thus be carefully assessed, including successful disease prevention or an inpatient undertreatment of certain diseases. The ongoing importance of population aging and its interaction with demand- and supply-side factors emphasizes the need for hospital structural reforms based on solid demographic evidence. This analysis informs policymakers about the quantitative impact of an aging population on hospital care utilization. In particular, future hospital planning by the federal states must consider the central role of population aging in shaping inpatient care needs.

## Supplementary Information

Supplementary Material 1: Table showing the case selection of the G-DRG Statistic by exclusion criterion and survey year.

Supplementary Material 2: Sex-specific figures illustrating contributions of changing age-specific hospitalization risks to THR changes by disease categories.

Supplementary Material 3: Results table showing contributions of changing age-specific hospitalization risks to THR changes by the most frequent disease categories.

Supplementary Material 4: Results table showing contributions of changing age-specific hospitalization risks to THR changes by all disease categories.

Supplementary Material 5: Figure comparing the development of hospital case numbers in Germany with the G-DRG analysis sample of the current study.

## Data Availability

This study is based on data from the German Diagnosis-Related Groups (G-DRG) Statistic (https://doi.org/10.21242/23141.2005.00.00.1.1.0 to 10.21242/23141.2021.00.00.1.1.0) and the Human Mortality Database from the years 2005 to 2021. The data analyzed in this study are openly accessible, and the results are fully reproducible with the codes and data available under: 10.17605/OSF.IO/W8ZJX.

## Abbreviations

G-DRG: German Diagnosis-Related Groups
ICD-10-GM: 10th revision of the International Classification of Diseases, German Modification
InEK: Institute for the Hospital Remuneration System
KHG: Act on the Economic Security of Hospitals and the Regulation of Hospital Nursing Rates
THR: Total Hospitalization Rate

## References

[1] Nowossadeck E, Prütz F, Teti A. Population change and the burden of hospitalization in Germany 2000–2040: decomposition analysis and projection. PLoS ONE. 2020;15:e0243322. 10.1371/journal.pone.0243322. 33306705 10.1371/journal.pone.0243322 PMC7732063

[2] Schoffer O, Schriefer D, Werblow A, Gottschalk A, Peschel P, Liang LA, et al. Modelling the effect of demographic change and healthcare infrastructure on the patient structure in German hospitals – a longitudinal national study based on official hospital statistics. BMC Health Serv Res. 2023;23:1081. 10.1186/s12913-023-10056-y. 37821860 10.1186/s12913-023-10056-y PMC10566170

[3] Federal Statistical Office. Einrichtungen, Betten und Patientenbewegung. Krankenhäuser. 2025. https://www.destatis.de/DE/Themen/Gesellschaft-Umwelt/Gesundheit/Krankenhauser/Tabellen/gd-krankenhaeuser-jahre.html. Accessed 3 Nov 2025.

[4] Federal Statistical Office. Bevölkerung: Deutschland, Stichtag. 2026. https://www-genesis.destatis.de/datenbank/online/table/12411-0001/table-toolbar. Accessed 22 Feb 2026.

[5] FürstenbergT, Laschat M, Zich K, Klein S, Gierling P, Nolting H-D, et al. G-DRG-Begleitforschung gemäß § 17b Abs. 8 KHG. Endbericht des zweiten Forschungszyklus (2006 bis 2008). Untersuchung im Auftrag des deutschen DRG-Instituts (InEK). 2013. https://www.gkv-spitzenverband.de/media/dokumente/krankenversicherung_1/krankenhaeuser/drg/drg_begleitforschung/DRG_Begleitforschung_Endbericht_2_Zyklus_2006_-_2008_2011_06.pdf. Accessed 3 Nov 2025.

[6] AugurzkyB, Gülker R, Mennicken R, Felder S, Meyer S, Wasem J, et al. Mengenentwicklung und Mengensteuerung stationärer Leistungen. Endbericht. Forschungsprojekt im Auftrag des GKV-Spitzenverbandes. 2012. https://www.gkv-spitzenverband.de/media/dokumente/presse/pressekonferenzen_gespraeche/2012_2/120529_mengenentwicklung_krankenhausbereich/RWI-Gutachten_Mengenentwicklung_2012_06_08_19832.pdf. Accessed 3 Nov 2025.

[7] BlumK, Offermanns M. Einflussfaktoren des Fallzahl- und Case Mix-Anstieges in deutschen Krankenhäusern. Gutachten des Deutschen Krankenhausinstituts (DKI) im Auftrag der Deutschen Krankenhausgesellschaft (DKG). 2012. https://www.dki.de/fileadmin/forschungsberichte/einflussfaktoren_des_fallzahl-_und_case_mix-anstiegs_in_deutschen_krankenhaeusern.pdf. Accessed 3 Nov 2025.

[8] Nowossadeck E. Population aging and hospitalization for chronic disease in Germany. Dtsch Arztebl Int. 2012;109:151–7. 10.3238/arztebl.2012.0151. 22461861 10.3238/arztebl.2012.0151 PMC3314234

[9] Klauber J, Brenner G, Augurzky B, editors. Mengendynamik: mehr Menge, mehr Nutzen? Stuttgart: Schattauer; 2013.

[10] Schreyögg, Jonas, Bäuml, Matthias, Busse, Reinhard, Geissler, Alexander. Forschungsauftrag zur Mengenentwicklung nach § 17b Abs. 9 KHG, Endbericht. 2014. https://www.g-drg.de/datenbrowser-und-begleitforschung/begleitforschung-drg/forschungsauftrag-gem.-17b-abs.-9-khg. Accessed 3 Nov 2025.

[11] Nowossadeck E, Prütz F. Regionale Unterschiede der Entwicklung der Krankenhausbehandlungen: Effekt unterschiedlicher demografischer Trends? Bundesgesundheitsblatt Gesundheitsforschung Gesundheitsschutz. 2018;61:358–66. 10.1007/s00103-018-2695-1. 29374298 10.1007/s00103-018-2695-1

[12] Krämer J, Schreyögg J. Demand-side determinants of rising hospital admissions in Germany: the role of ageing. Eur J Health Econ. 2019;20:715–28. 10.1007/s10198-019-01033-6. 30739296 10.1007/s10198-019-01033-6 PMC6602979

[13] Krämer J, Schreyögg J. Messung des Einflusses einer alternden Bevölkerung und anderer nachfrageseitiger Determinanten auf die Inanspruchnahme stationärer Leistungen. In: Klauber J, Wasem J, Beivers A, Mostert C, Scheller-Kreinsen D, editors. Krankenhaus-Report 2025: Versorgung Hochbetagter. Berlin, Heidelberg: Springer; 2025;49–66. 10.1007/978-3-662-70947-4_3.

[14] Milstein R, Schreyögg J. Empirische Evidenz zu den Wirkungen der Einführung des G-DRG-Systems. In: Klauber J, Geraedts M, Friedrich J, Wasem J, Beivers A, editors. Krankenhaus-Report 2020. Berlin, Heidelberg: Springer Berlin Heidelberg; 2020;25–39. 10.1007/978-3-662-60487-8_2.

[15] Messerle R, Schreyögg J. System-wide Effects of Hospital Payment Scheme Reforms: The German Introduction of Diagnosis-Related Groups. 2022. https://www.hche.uni-hamburg.de/dokumente/research-papers/rp-26-messerle-schreyoegg-2022-drg.pdf. Accessed 3 Nov 2025.

[16] van Raalte AA, Basellini U, Camarda CG, Nepomuceno MR, Myrskylä M. The dangers of drawing cohort profiles from period data: a research note. Demography. 2023;60:1689–98. 10.1215/00703370-11067917. 37965885 10.1215/00703370-11067917 PMC10843689

[17] Hoem JM, Keiding N, Kulokari H, Natvig B, Barndorff-Nielsen O, Hilden J. The statistical theory of demographic rates: a review of current developments [with discussion and reply]. Scand J Stat. 1976;3:169–85.

[18] Kuan V, Denaxas S, Gonzalez-Izquierdo A, Direk K, Bhatti O, Husain S, et al. A chronological map of 308 physical and mental health conditions from 4 million individuals in the English National Health Service. Lancet Digit Health. 2019;1:e63-77. 10.1016/S2589-7500(19)30012-3. 31650125 10.1016/S2589-7500(19)30012-3 PMC6798263

[19] Schelhase T. Statistische Krankenhausdaten: Diagnosedaten der Krankenhauspatienten 2022. In: Klauber J, Wasem J, Beivers A, Mostert C, Scheller-Kreinsen D, editors. Krankenhaus-Report 2024. Berlin, Heidelberg: Springer Berlin Heidelberg; 2024. p. 465–97. 10.1007/978-3-662-68792-5_22.

[20] Research Data Centres of the Federal Statistical Office and the Statistical Offices of the Federal States. Diagnosis-Related Groups Statistic. 10.21242/23141.2005.00.00.1.1.0to10.21242/23141.2021.00.00.1.1.0.

[21] Research Data Centres of the Federal Statistical Office and the Statistical Offices of the Federal States. Diagnosis-Related Groups Statistic. https://www.forschungsdatenzentrum.de/en/health/drg. Accessed 9 Jun 2025.

[22] Research Data Centres of the Statistical Offices of the Federation and the Federal States. Metadata Report. Part I: General and methodological information on the Diagnosis-Related Groups Statistic (DRG) (EVAS number: 23141). Version 2. 2019. https://www.forschungsdatenzentrum.de/sites/default/files/drg_on-site_mdr-stat_en.pdf. Accessed 6 Jun 2025.

[23] Max Planck Institute for Demographic Research, University of California, Berkeley, French Institute for Demographic Studies. HMD. Human Mortality Database. Germany, total population, period data, Exposure to risk (period 1x1) (1990–2020) (age-interval x year-interval: 1x1). Last modified: 03 June 2022. https://www.mortality.org/Country/Country?cntr=DEUTNP. Accessed 14 Mar 2024.

[24] Max Planck Institute for Demographic Research, University of California, Berkeley, French Institute for Demographic Studies. HMD. Human Mortality Database. Germany, total population, period data, population size (abridged) (1990–2021) (age-interval x year-interval: 1x1). Last modified: 03 June 2022. https://www.mortality.org/Country/Country?cntr=DEUTNP. Accessed 14 Mar 2024.

[25] Research Data Centres of the Federal Statistical Office and the Statistical Offices of the Federal States. Regulations on the analysis of microdata in the Research Data Centres of the Federal Statistical Office and the Statistical Offices of the Federal States (RDC). Effective January 14th 2022. 2022. https://www.forschungsdatenzentrum.de/sites/default/files/RDC_regulations-microdata_2022.pdf. Accessed 3 Nov 2025.

[26] OECD. Hospital discharge rates. https://www.oecd.org/en/data/indicators/hospital-discharge-rates.html. Accessed 6 Nov 2025.

[27] Federal Statistical Office. Zensus 2011. Bevölkerungsstand. https://www.destatis.de/DE/Themen/Gesellschaft-Umwelt/Bevoelkerung/Bevoelkerungsstand/Glossar/zensus2011.html. Accessed 29 Dec 2024.

[28] Kitagawa EM. Components of a difference between two rates. J Am Stat Assoc. 1955;50:1168–94. 10.1080/01621459.1955.10501299.

[29] Stucki M. Factors related to the change in Swiss inpatient costs by disease: a 6-factor decomposition. Eur J Health Econ. 2021;22:195–221. 10.1007/s10198-020-01243-3. 33433763 10.1007/s10198-020-01243-3 PMC7881977

[30] Dieleman JL, Squires E, Bui AL, Campbell M, Chapin A, Hamavid H, et al. Factors associated with increases in US health care spending, 1996-2013. JAMA. 2017;318:1668–78. 10.1001/jama.2017.15927. 29114831 10.1001/jama.2017.15927 PMC5818797

[31] R Core Team. _R: A Language and Environment for Statistical Computing_. 2025. https://www.r-project.org/. Accessed 2 May 2024.

[32] Federal Statistical Office. Population: Germany, reference date, age. 2026. https://www-genesis.destatis.de/datenbank/online/url/1786096f. Accessed 24 Feb 2026.

[33] Max Planck Institute for Demographic Research, University of California, Berkeley, French Institute for Demographic Studies. HMD. Human Mortality Database. Germany, Life expectancy at birth (1990–2020) (period, 1x1). Last modified: 03 June 2022. https://www.mortality.org/Country/Country?cntr=DEUTNP. Accessed 14 Mar 2024.

[34] Goujon A, Jacobs-Crisioni C, Natale F, Lavalle C, editors. The Demographic Landscape of Eu Territories: Challenges and Opportunities in Diversely Ageing Regions [scientific Report]. European Union; 2021. https://data.europa.eu/doi/10.2760/658945. Accessed 5 Sep 2025.

[35] United Nations Department of Economic and Social Affairs, Population Division. World Population Prospects 2022: Summary of Results. New York: United Nations; 2022.

[36] German Hospital Federation. Bestandsaufnahme zur Krankenhausplanung und Investitionsfinanzierung in den Bundesländern 2022. 2022. https://www.dkgev.de/fileadmin/default/Mediapool/2_Themen/2.3_Versorgung-Struktur/2.3.1_Planung/2022_DKG_Bestandsaufnahme_KH-Planung_und_Investitionsfinanzierung.pdf. Accessed 25 Jun 2025.

[37] Vogel J, Letzgus P, Geissler A. Paradigmenwechsel in der Krankenhausplanung – hin zu Leistungs-, Bedarfs- und Qualitätsorientierung für einen höheren Patientennutzen. In: Klauber J, Geraedts M, Friedrich J, Wasem J, Beivers A, editors. Krankenhaus-Report 2020: Finanzierung und Vergütung am Scheideweg. Berlin, Heidelberg: Springer; 2020;327–58. 10.1007/978-3-662-60487-8_18.

[38] Federal Ministry of Health (BMG). Eckpunktepapier Krankenhausreform. 2023. https://www.bundesgesundheitsministerium.de/fileadmin/Dateien/3_Downloads/K/Krankenhausreform/Eckpunktepapier_Krankenhausreform_final.pdf. Accessed 3 Nov 2025.

[39] Federal Ministry of Health (BMG). Entwurf eines Gesetzes zur Anpassung der Krankenhausreform (Krankenhausreformanpassungsgesetz – KHAG). 2025. https://www.bundesgesundheitsministerium.de/fileadmin/Dateien/3_Downloads/Gesetze_und_Verordnungen/GuV/K/RefE_Krankenhausreformanpassungsgesetz_-_KHAG.pdf. Accessed 23 Sep 2025.

[40] Plass D, Vos T, Hornberg C, Scheidt-Nave C, Zeeb H, Krämer A. Trends in Disease Burden in Germany. Dtsch Ärztebl Int. 2014. 10.3238/arztebl.2014.0629. 25316518 10.3238/arztebl.2014.0629 PMC4199248

[41] Porst M, Von Der Lippe E, Leddin J, Anton A, Wengler A, Breitkreuz J, et al. The burden of disease in Germany at the national and regional level—results in terms of disability-adjusted life years (DALY) from the BURDEN 2020 study. Dtsch Arztebl Int. 2022. 10.3238/arztebl.m2022.0314. 36350160 10.3238/arztebl.m2022.0314 PMC9902892

[42] Simon M. Das Gesundheitssystem in Deutschland: eine Einführung in Struktur und Funktionsweise. 7. überarbeitete und erweiterte Auflage. Bern: Hogrefe; 2021.

[43] Federal Ministry of Health (BMG). Weiterentwicklung der onkologischen Versorgungsstrukturen. 2025. https://www.bundesgesundheitsministerium.de/themen/praevention/nationaler-krebsplan/handlungsfelder/handlungsfeld-2. Accessed 24 Feb 2026.

[44] Rombey T, Eckhardt H, Felgner S, Dreger M, Campione A, Ermann H, et al. Mind the (research) gap: a retrospective observational study on the utilization of new medical technologies and related research activities in German hospitals. Health Res Policy Syst. 2025;23:72. 10.1186/s12961-025-01342-8. 40448162 10.1186/s12961-025-01342-8 PMC12124022

[45] Federal Statistical Office. 2,0 % mehr stationäre Krankenhausbehandlungen im Jahr 2024. https://www.destatis.de/DE/Presse/Pressemitteilungen/2025/11/PD25_398_231.html. Accessed 22 Feb 2026.

[46] Hentschker C, Mostert C, Klauber J. Auswirkungen der Covid-19-Pandemie im Krankenhaus: Fallzahlentwicklung und Charakteristika der Covid-19-Patienten. In: Klauber J, Wasem J, Beivers A, Mostert C, editors. Krankenhaus-Report 2023: Schwerpunkt: Personal. Berlin, Heidelberg: Springer; 2023;305–23. 10.1007/978-3-662-66881-8_19.

[47] Tillmanns H, Jäckel D. Entwicklung der Ambulantisierung. In: Klauber J, Wasem J, Beivers A, Mostert C, Scheller-Kreinsen D, editors. Krankenhaus-Report 2024. Berlin, Heidelberg: Springer Berlin Heidelberg; 2024;225–68. 10.1007/978-3-662-68792-5_12.

[48] Dittmann H. Das Krankenhausstrukturgesetz: Hehre Ziele – Ernüchternde Umsetzung. Gesundheitsokonomie Qual. 2018;23:29–34. 10.1055/s-0043-109524.

[49] Federal Statistical Office. Deaths: Germany, years, sex, age years. 2026. https://www-genesis.destatis.de/datenbank/online/url/b4d6d976. Accessed 24 Feb 2026.

