# Supplementary material for: Population aging and changing hospitalization risks in Germany: a decomposition of changes in inpatient cases, 2005–2021
Source: BMC Public Health. 2026 Apr 30;26:1437. doi: 10.1186/s12889-026-27522-x (PMC13135272; doi:10.1186/s12889-026-27522-x)
Supplement: Supplementary file 1 — Table showing the case selection of the G-DRG Statistic by exclusion criterion and survey year. [file 12889_2026_27522_MOESM1_ESM.pdf]

Case selection of the G-DRG Statistic by survey year (2005-2021)

Table S1 Case selection of the G-DRG Statistic by exclusion criterion and survey year

**Year**	**2005**		**2006**		**2007**	
Original case number (%)	16,071,846	(100.00)	16,230,407	(100.00)	16,600,472	(100.00)
Day cases (%)	457,917	(2.85)	453,165	(2.79)	462,899	(2.79)
Cases transferred to another hospital (%)	449,444	(2.80)	446,671	(2.75)	450,863	(2.72)
Patients with a foreign or unknown residence (%)	175,631	(1.09)	131,298	(0.81)	113,897	(0.69)
Missing values in the variable age (%)	3	(<0.01)	7	(<0.01)	2	(<0.01)
Missing values in the variable sex (%)	924	(0.01)	1,090	(0.01)	823	(<0.01)
Error DRG cases (%)	48,139	(0.30)	46,254	(0.28)	45,014	(0.27)
Missing values in the variable cause of discharge (%)	644	(<0.01)	474	(<0.01)	406	(<0.01)
Confidential cases (%)	11	(<0.01)	13	(<0.01)	63	(<0.01)
Analysis sample (%)	14,939,133	(92.95)	15,151,435	(93.35)	15,526,505	(93.53)
**Year**	**2008**		**2009**		**2010**	
Original case number (%)	16,924,180	(100.00)	17,191,063	(100.00)	17,434,400	(100.00)
Day cases (%)	470,480	(2.78)	481,526	(2.80)	494,893	(2.84)
Cases transferred to another hospital (%)	454,289	(2.68)	460,866	(2.68)	474,259	(2.72)
Patients with a foreign or unknown residence (%)	130,347	(0.77)	102,182	(0.59)	96,786	(0.56)
Missing values in the variable age (%)	6	(<0.01)	7	(<0.01)	0	(<0.01)
Missing values in the variable sex (%)	595	(<0.01)	411	(<0.01)	507	(<0.01)
Error DRG cases (%)	44,264	(0.26)	45,441	(0.26)	44,316	(0.25)
Missing values in the variable cause of discharge (%)	236	(<0.01)	0	(0.00)	0	(0.00)
Confidential cases (%)	47	(<0.01)	106	(<0.01)	104	(<0.01)
Analysis sample (%)	15,823,916	(93.50)	16,100,524	(93.66)	16,323,535	(93.63)

Table S1 (continued)

Year	2011		2012		2013	
Original case number (%)	17,708,910	(100.00)	17,976,447	(100.00)	18,133,711	(100.00)
Day cases (%)	503,524	(2.84)	513,774	(2.86)	510,720	(2.82)
Cases transferred to another hospital (%)	482,662	(2.73)	488,373	(2.72)	491,153	(2.71)
Patients with a foreign or unknown residence (%)	95,139	(0.54)	79,366	(0.44)	78,322	(0.43)
Missing values in the variable age (%)	10	(<0.01)	4	(<0.01)	8	(<0.01)
Missing values in the variable sex (%)	323	(<0.01)	1,372	(0.01)	308	(<0.01)
Error DRG cases (%)	45,151	(0.25)	45,715	(0.25)	45,536	(0.25)
Missing values in the variable cause of discharge (%)	0	(0.00)	0	(0.00)	0	(0.00)
Confidential cases (%)	43	(<0.01)	21	(<0.01)	44	(<0.01)
Analysis sample (%)	16,582,058	(93.64)	16,847,822	(93.72)	17,007,620	(93.79)
**Year**	**2014**		**2015**		**2016**	
Original case number (%)	18,531,819	(100.00)	18,665,238	(100.00)	18,961,650	(100.00)
Day cases (%)	526,651	(2.84)	534,734	(2.86)	549,681	(2.90)
Cases transferred to another hospital (%)	511,577	(2.76)	520,117	(2.79)	535,209	(2.82)
Patients with a foreign or unknown residence (%)	83,330	(0.45)	85,955	(0.46)	97,765	(0.52)
Missing values in the variable age (%)	10	(<0.01)	33	(<0.01)	18	(<0.01)
Missing values in the variable sex (%)	264	(<0.01)	261	(<0.01)	151	(<0.01)
Error DRG cases (%)	44,420	(0.24)	42,080	(0.23)	41,400	(0.22)
Missing values in the variable cause of discharge (%)	0	(0.00)	0	(0.00)	0	(0.00)
Confidential cases (%)	40	(<0.01)	28	(<0.01)	30	(<0.01)
Analysis sample (%)	17,365,527	(93.71)	17,482,030	(93.66)	17,737,396	(93.54)

Table S1 (continued)

Year	2017		2018		2019	
Original case number (%)	18,901,222	(100.00)	18,754,571	(100.00)	18,825,654	(100.00)
Day cases (%)	532,564	(2.82)	506,738	(2.70)	492,516	(2.62)
Cases transferred to another hospital (%)	541,547	(2.87)	533,865	(2.85)	538,477	(2.86)
Patients with a foreign or unknown residence (%)	81,804	(0.43)	84,660	(0.45)	84,643	(0.45)
Missing values in the variable age (%)	11	(<0.01)	19	(<0.01)	22	(<0.01)
Missing values in the variable sex (%)	1,006	(0.01)	2,632	(0.01)	0	(0.00)
Error DRG cases (%)	440	(<0.01)	202	(<0.01)	309	(<0.01)
Missing values in the variable cause of discharge (%)	0	(0.00)	0	(0.00)	0	(0.00)
Confidential cases (%)	35	(<0.01)	37	(<0.01)	36	(<0.01)
Analysis sample (%)	17,743,815	(93.88)	17,626,418	(93.98)	17,709,651	(94.07)
**Year**	**2020**		**2021**		**2005–2021**	
Original case number (%)	16,359,312	(100.00)	16,233,822	(100.00)	299,504,724	(100.00)
Day cases (%)	411,308	(2.51)	381,731	(2.35)	8,284,821	(2.77)
Cases transferred to another hospital (%)	486,210	(2.97)	497,053	(3.06)	8,362,635	(2.79)
Patients with a foreign or unknown residence (%)	60,598	(0.37)	68,034	(0.42)	1,649,757	(0.55)
Missing values in the variable age (%)	15	(<0.01)	18	(<0.01)	193	(<0.01)
Missing values in the variable sex (%)	0	(0.00)	0	(0.00)	10,667	(<0.01)
Error DRG cases (%)	255	(<0.01)	237	(<0.01)	539,173	(0.18)
Missing values in the variable cause of discharge (%)	0	(0.00)	0	(0.00)	1,760	(0.00)
Confidential cases (%)	117	(<0.01)	134	(<0.01)	909	(<0.01)
Analysis sample (%)	15,400,809	(94.14)	15,286,615	(94.17)	280,654,809	(93.71)
