# Supplementary material for: Population aging and changing hospitalization risks in Germany: a decomposition of changes in inpatient cases, 2005–2021
Source: BMC Public Health. 2026 Apr 30;26:1437. doi: 10.1186/s12889-026-27522-x (PMC13135272; doi:10.1186/s12889-026-27522-x)
Supplement: Supplementary file 2 — Sex-specific figures illustrating contributions of changing age-specific hospitalization risks to THR changes by disease categories. [file 12889_2026_27522_MOESM2_ESM.pdf]

Contributions of changing age-specific hospitalization risks to THR changes by disease categories and sex

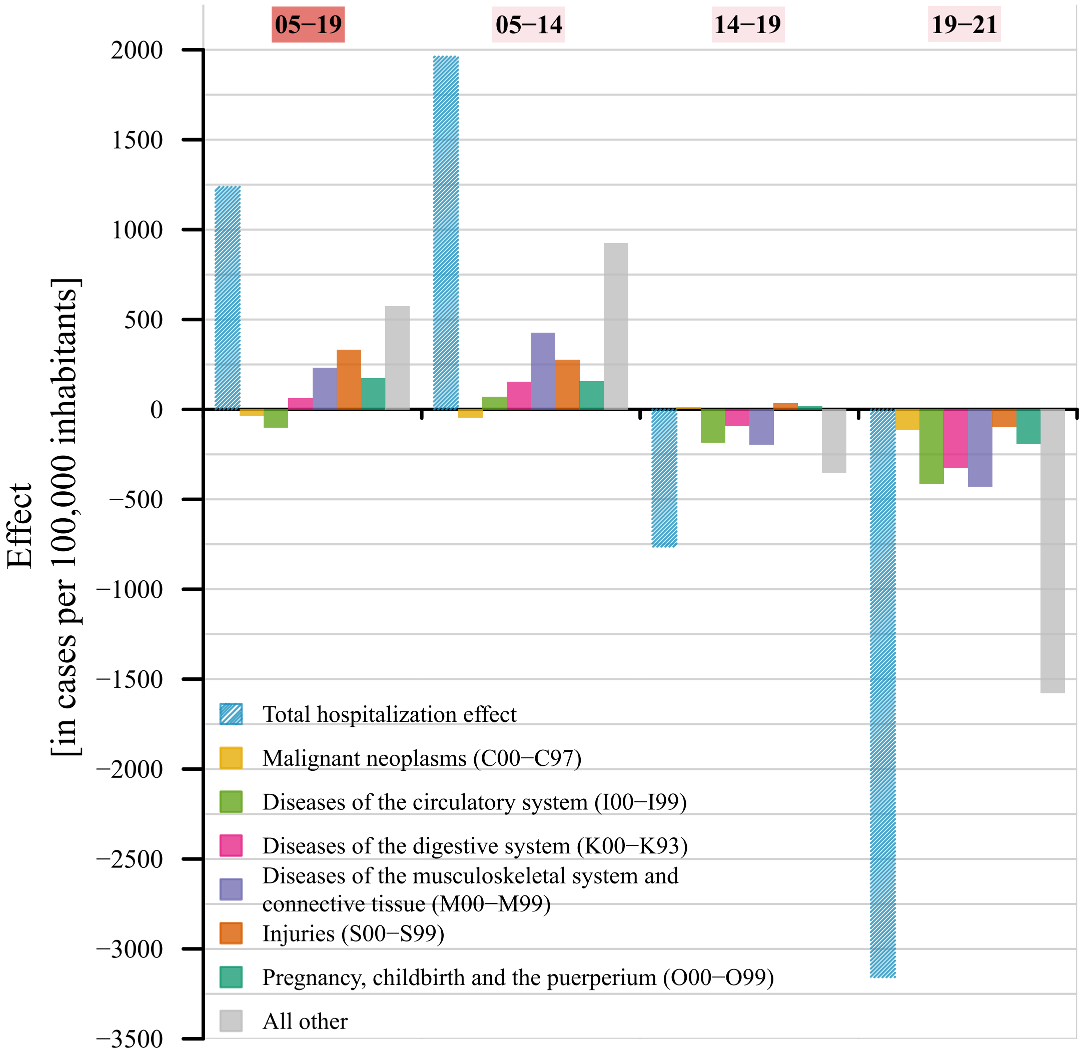

Fig. S1 Stratification of the hospitalization risk effect by disease category, women – the five most prevalent disease categories are presented for the time periods 2005-2019 (highlighted in red), 2005-2014, 2014-2019, and 2019-2021 (all highlighted in light rose): diseases of the circulatory system (ICD-10-GM: I00-I99), malignant neoplasms (C00-C97), diseases of the digestive system (K00-K93), diseases of the musculoskeletal system and connective tissue (M00-M99), injuries (S00-S99), as well as the disease category pregnancy, childbirth, and the puerperium (O00-O99), and all other – *ICD-10-GM* 10th revision of the International Classification of Diseases, German Modification

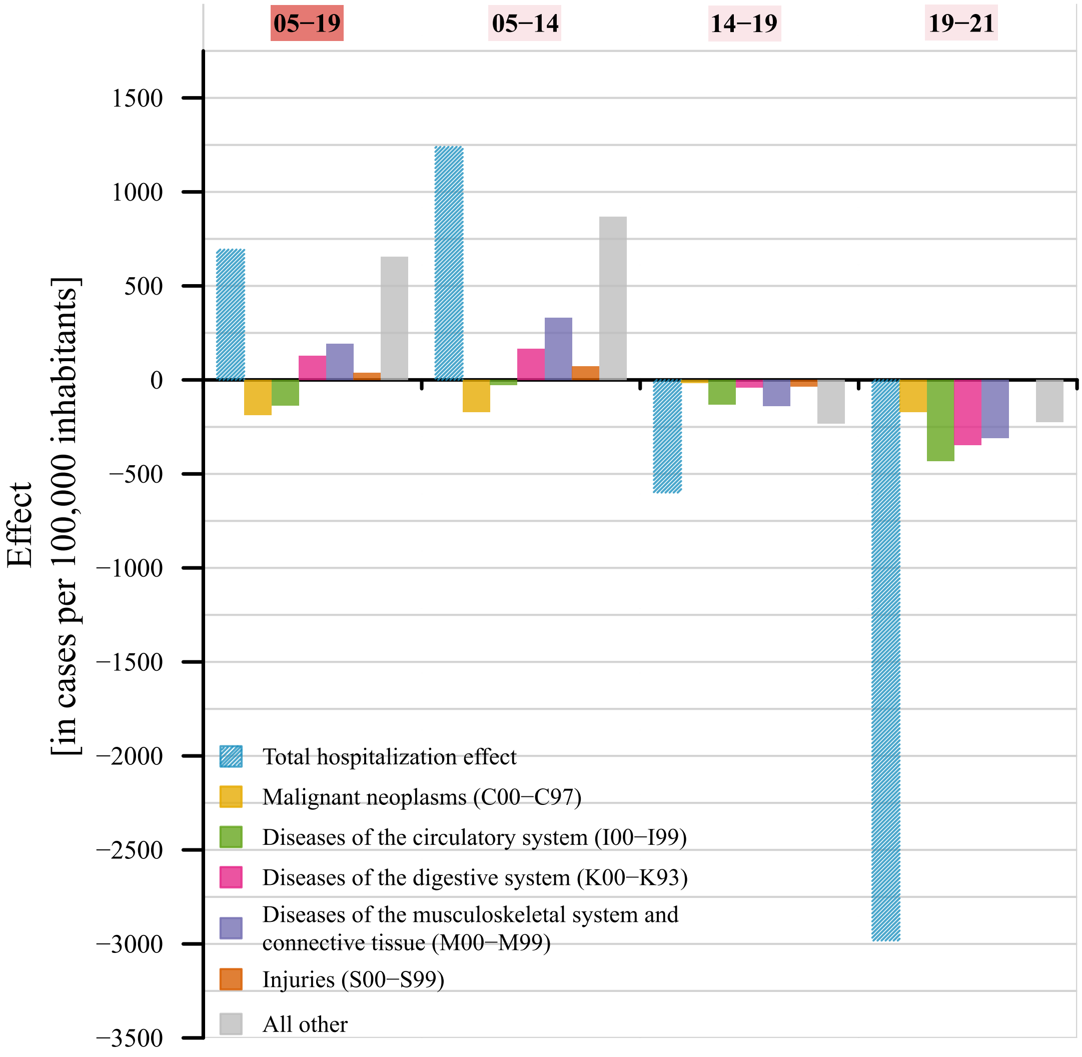

Fig. S2 Stratification of the hospitalization risk effect by disease category, men – the five most prevalent disease categories are presented for the time periods 2005-2019 (highlighted in red), 2005-2014, 2014-2019, and 2019-2021 (all highlighted in light rose): diseases of the circulatory system (ICD-10-GM: I00-I99), malignant neoplasms (C00-C97), diseases of the digestive system (K00-K93), diseases of the musculoskeletal system and connective tissue (M00-M99), injuries (S00-S99), and all other – *ICD-10-GM* 10th revision of the International Classification of Diseases, German Modification
