# Supplementary material for: Population aging and changing hospitalization risks in Germany: a decomposition of changes in inpatient cases, 2005–2021
Source: BMC Public Health. 2026 Apr 30;26:1437. doi: 10.1186/s12889-026-27522-x (PMC13135272; doi:10.1186/s12889-026-27522-x)
Supplement: Supplementary file 3 — Results table showing contributions of changing age-specific hospitalization risks to THR changes by the most frequent disease categories. [file 12889_2026_27522_MOESM3_ESM.pdf]

## Stratification of the hospitalization risk effect by the most frequent diagnostic categories

**Table S2** Stratification of the hospitalization risk effect by disease category, total population and by sex

| Period | | Total population | | Women | | Men | |
|---|---|---|---|---|---|---|---|
| | | (cases per 100,000 inhabitants) | (%) | (cases per 100,000 inhabitants) | (%) | (cases per 100,000 inhabitants) | (%) |
| **2005 – 2019** | Hospitalization risk effect | 1,041 | 100.0 | 1,238 | 100.0 | 694 | 100.0 |
| | Malignant neoplasms (C00-C97) | -81 | -7.8 | -37 | -3.0 | -187 | -27.0 |
| | Diseases of the circulatory system (I00-I99) | -88 | -8.5 | -101 | -8.2 | -137 | -19.7 |
| | Diseases of the digestive system (K00-K93) | 106 | 10.2 | 62 | 5.0 | 130 | 18.7 |
| | Diseases of the musculoskeletal system and connective tissue (M00-M99) | 204 | 19.6 | 233 | 18.8 | 193 | 27.8 |
| | Pregnancy, childbirth, and the puerperium (O00-O99) | 147 | 14.1 | 332 | 26.8 | 0 | 0.0 |
| | Injuries (S00-S99) | 90 | 8.6 | 174 | 14.1 | 38 | 5.5 |
| | All other | 663 | 63.7 | 574 | 46.4 | 656 | 94.5 |
| **2005 – 2014** | Hospitalization risk effect | 1,666 | 100.0 | 1,962 | 100.0 | 1,241 | 100.0 |
| | Malignant neoplasms (C00-C97) | -86 | -5.9 | -46 | -2.7 | -172 | -15.4 |
| | Diseases of the circulatory system (I00-I99) | 45 | 3.1 | 70 | 4.2 | -27 | -2.4 |
| | Diseases of the digestive system (K00-K93) | 167 | 11.5 | 154 | 9.2 | 166 | 14.8 |
| | Diseases of the musculoskeletal system and connective tissue (M00-M99) | 371 | 25.4 | 426 | 25.4 | 331 | 29.6 |
| | Pregnancy, childbirth, and the puerperium (O00-O99) | 134 | 9.2 | 276 | 16.5 | 0 | 0.0 |
| | Injuries (S00-S99) | 102 | 6.1 | 156 | 8.0 | 73 | 5.9 |
| | All other | 932 | 55.9 | 926 | 47.2 | 870 | 70.1 |

**Table S2** (continued)

| Period | | Total population | | Women | | Men | |
|---|---|---|---|---|---|---|---|
| | | (cases per 100,000 inhabitants) | (%) | (cases per 100,000 inhabitants) | (%) | (cases per 100,000 inhabitants) | (%) |
| **2014 – 2019** | Hospitalization risk effect | -674 | 100.0 | -763 | 100.0 | -599 | 100.0 |
| | Malignant neoplasms (C00-C97) | 5 | -0.7 | 11 | -1.4 | -17 | 2.8 |
| | Diseases of the circulatory system (I00-I99) | -151 | 22.4 | -184 | 24.1 | -132 | 22.0 |
| | Diseases of the digestive system (K00-K93) | -64 | 9.5 | -92 | 12.1 | -40 | 6.7 |
| | Diseases of the musculoskeletal system and connective tissue (M00-M99) | -171 | 25.4 | -197 | 25.8 | -140 | 23.4 |
| | Pregnancy, childbirth, and the puerperium (O00-O99) | 1 | -0.1 | 35 | -4.6 | 0 | 0.0 |
| | Injuries (S00-S99) | -13 | 1.9 | 18 | -2.4 | -37 | 6.2 |
| | All other | -280 | 41.5 | -354 | 46.4 | -234 | 39.1 |
| **2019 – 2021** | Hospitalization risk effect | -3,066 | 100.0 | -3,158 | 100.0 | -2,982 | 100.0 |
| | Malignant neoplasms (C00-C97) | -141 | 4.6 | -116 | 3.7 | -170 | 5.7 |
| | Diseases of the circulatory system (I00-I99) | -423 | 13.8 | -416 | 13.2 | -433 | 14.5 |
| | Diseases of the digestive system (K00-K93) | -337 | 11.0 | -327 | 10.4 | -348 | 11.7 |
| | Diseases of the musculoskeletal system and connective tissue (M00-M99) | -370 | 12.1 | -429 | 13.6 | -310 | 10.4 |
| | Pregnancy, childbirth, and the puerperium (O00-O99) | -50 | 1.6 | -97 | 3.1 | 0 | 0.0 |
| | Injuries (S00-S99) | -210 | 6.8 | -193 | 6.1 | -226 | 7.6 |
| | All other | -1535 | 50.1 | -1580 | 50.0 | -1496 | 50.2 |

The five most prevalent disease categories are presented for the time periods 2005-2019, 2005-2014, 2014-2019, and 2019-2021: diseases of the circulatory system (ICD-10-GM: I00-I99), malignant neoplasms (C00-C97), diseases of the digestive system (K00-K93), diseases of the musculoskeletal system and connective tissue (M00-M99), injuries (S00-S99), as well as the disease category pregnancy, childbirth, and the puerperium (O00-O99), and all other – the stratification of the hospitalization risk effects by all disease categories is shown in Additional file 2: Table S1 – *ICD-10-GM* 10th revision of the International Classification of Diseases, German Modification
