# Supplementary material for: Population aging and changing hospitalization risks in Germany: a decomposition of changes in inpatient cases, 2005–2021
Source: BMC Public Health. 2026 Apr 30;26:1437. doi: 10.1186/s12889-026-27522-x (PMC13135272; doi:10.1186/s12889-026-27522-x)
Supplement: Supplementary file 4 — Results table showing contributions of changing age-specific hospitalization risks to THR changes by all disease categories. [file 12889_2026_27522_MOESM4_ESM.pdf]

Stratification of the hospitalization risk effect by all diagnostic categories

Table S3: Stratification of the hospitalization risk effects by all disease categories

**Hospitalization effect by ICD main chapter (ICD-10-GM)**	**2005-2019**					
	**Total population**		**Women (%)**		**Men (%)**	
	**(cases per 100,000 inhabi-tants)**	**(%)**	**(cases per 100,000 inhabi-tants)**	**(%)**	**(cases per 100,000 inhabi-tants)**	**(%)**
Total hospitalization risk effect	1041	100	1238	100	694	100
A00-B99 Certain infectious and parasitic diseases	183	17.6	179	14	182	26.2
C00-C97 Malignant neoplasms	-81	-7.8	-37	-3	-187	-26.9
D00-D48 In situ neoplasms, benign neoplasms, neoplasms of uncertain or unknown behaviour, Diseases of the blood and haematopoietic organs and certain disorders involving the immune system	-1	-0.1	-47	-4	43	6.2
E00-E90 Endocrine, nutritional and metabolic diseases	-16	-1.5	-43	-3	16	2.3
F00-F99 Mental and behavioural disorders	-17	-1.6	-17	-1	-16	-2.3
G00-G99 Diseases of the nervous system	-22	-2.1	53	4	-109	-15.7
H00-H95 Diseases of the eye and ocular appendages and diseases of the ear and mastoid process	3	0.3	-10	-1	17	2.4
I00-I99 Diseases of the circulatory system	-88	-8.5	-101	-8	-137	-19.7
J00-J99 Diseases of the respiratory system	69	6.6	92	7	1	0.1
K00-K93 Diseases of the digestive system	106	10.2	62	5	130	18.7
L00-L99 Diseases of the skin and subcutis	65	6.2	52	4	76	11.0
M00-M99 Diseases of the musculoskeletal system and connective tissue	204	19.6	233	19	193	27.8
N00-N99 Diseases of the urogenital system	143	13.7	54	4	210	30.3
O00-O99 Pregnancy, childbirth and puerperium	147	14.1	332	27	0	0.0
P00-P96 Certain states originating in the perinatal period	24	2.3	21	2	27	3.9
Q00-Q99 Congenital malformations, deformities and chromosomal anomalies	-1	-0.1	-5	0	3	0.4
R00-R99 Symptoms and abnormal clinical and laboratory findings not elsewhere classified	252	24.2	275	22	222	32.0
S00-S99 Injuries	90	8.6	174	14	38	5.5
T00-T98 Poisoning, and certain other consequences of external causes	16	1.5	4	0	26	3.7
Z00-Z99 Factors influencing health status and leading to use of health services	-35	-3.4	-33	-3	-41	-5.9
U00-U99 key for special purposes						

Table S3 (continued)

Hospitalization effect by ICD main chapter (ICD-10-GM)	2005-2014					
	Total population		Women		Men	
	(cases per 100,000 inhabi-tants)	(%)	(cases per 100,000 inhabi-tants)	(%)	(cases per 100,000 inhabi-tants)	(%)
Total hospitalization risk effect	1666	100	1963	100	1242	100
A00-B99 Certain infectious and para-sitic diseases	182	10.9	190	9.7	171	13.8
C00-C97 Malignant neoplasms	-86	-5.2	-46	-2.3	-172	-13.8
D00-D48 In situ neoplasms, benign neoplasms, neoplasms of uncertain or unknown behaviour, Diseases of the blood and haematopoietic organs and certain disorders involving the im-mune system	11	0.7	-15	-0.8	34	2.7
E00-E90 Endocrine, nutritional and metabolic diseases	-1	-0.1	-18	-0.9	20	1.6
F00-F99 Mental and behavioural dis-orders	9	0.5	4	0.2	15	1.2
G00-G99 Diseases of the nervous system	37	2.2	94	4.8	-30	-2.4
H00-H95 Diseases of the eye and oc-ular appendages and diseases of the ear and mastoid process	22	1.3	19	1.0	27	2.2
I00-I99 Diseases of the circulatory system	45	2.7	70	3.6	-27	-2.2
J00-J99 Diseases of the respiratory system	56	3.4	64	3.3	16	1.3
K00-K93 Diseases of the digestive system	167	10.0	154	7.8	166	13.4
L00-L99 Diseases of the skin and subcutis	73	4.4	60	3.1	87	7.0
M00-M99 Diseases of the musculo-skeletal system and connective tissue	371	22.3	426	21.7	331	26.7
N00-N99 Diseases of the urogenital system	122	7.3	89	4.5	138	11.1
O00-O99 Pregnancy, childbirth and puerperium	134	8.0	276	14.1	0	0.0
P00-P96 Certain states originating in the perinatal period	27	1.6	24	1.2	30	2.4
Q00-Q99 Congenital malformations, deformities and chromosomal anoma-lies	3	0.2	0	0.0	6	0.5
R00-R99 Symptoms and abnormal clinical and laboratory findings not elsewhere classified	340	20.4	377	19.2	297	23.9
S00-S99 Injuries	102	6.1	156	7.9	73	5.9
T00-T98 Poisoning, and certain other consequences of external causes	61	3.7	48	2.4	71	5.7
Z00-Z99 Factors influencing health status and leading to use of health services	-9	-0.5	-9	-0.5	-11	-0.9
U00-U99 key for special purposes						

Table S3 (continued)

Hospitalization effect by ICD main chapter (ICD-10-GM)	2014-2019					
	Total population		Women		Men	
	(cases per 100,000 inhabi-tants)	(%)	(cases per 100,000 inhabi-tants)	(%)	(cases per 100,000 inhabi-tants)	(%)
Total hospitalization risk effect	-672	100	-763	100	-600	100
A00-B99 Certain infectious and parasitic diseases	0	0.0	-12	1.6	10	-1.7
C00-C97 Malignant neoplasms	5	-0.7	11	-1.4	-17	2.8
D00-D48 In situ neoplasms, benign neoplasms, neoplasms of uncertain or unknown behaviour, Diseases of the blood and haematopoietic organs and certain disorders involving the immune system	-13	1.9	-34	4.5	8	-1.3
E00-E90 Endocrine, nutritional and metabolic diseases	-18	2.7	-27	3.5	-7	1.2
F00-F99 Mental and behavioural disorders	-26	3.9	-20	2.6	-32	5.3
G00-G99 Diseases of the nervous system	-60	8.9	-41	5.4	-83	13.8
H00-H95 Diseases of the eye and ocular appendages and diseases of the ear and mastoid process	-19	2.8	-28	3.7	-9	1.5
I00-I99 Diseases of the circulatory system	-151	22.5	-184	24.1	-132	22.0
J00-J99 Diseases of the respiratory system	20	-3.0	36	-4.7	-10	1.7
K00-K93 Diseases of the digestive system	-64	9.5	-92	12.1	-40	6.7
L00-L99 Diseases of the skin and subcutis	-9	1.3	-8	1.0	-12	2.0
M00-M99 Diseases of the musculoskeletal system and connective tissue	-171	25.4	-197	25.8	-140	23.3
N00-N99 Diseases of the urogenital system	19	-2.8	-37	4.8	70	-11.7
O00-O99 Pregnancy, childbirth and puerperium	1	-0.1	35	-4.6	0	0.0
P00-P96 Certain states originating in the perinatal period	-4	0.6	-5	0.7	-4	0.7
Q00-Q99 Congenital malformations, deformities and chromosomal anomalies	-4	0.6	-5	0.7	-3	0.5
R00-R99 Symptoms and abnormal clinical and laboratory findings not elsewhere classified	-90	13.4	-103	13.5	-79	13.2
S00-S99 Injuries	-13	1.9	18	-2.4	-37	6.2
T00-T98 Poisoning, and certain other consequences of external causes	-47	7.0	-46	6.0	-50	8.3
Z00-Z99 Factors influencing health status and leading to use of health services	-28	4.2	-24	3.1	-33	5.5
U00-U99 key for special purposes						

Table S3 (continued)

**Hospitalization effect by ICD main chapter (ICD-10-GM)**	**2019-2021**					
	**Total population**		**Women**		**Men**	
	**(cases per 100,000 inhabi-tants)**	**(%)**	**(cases per 100,000 inhabi-tants)**	**(%)**	**(cases per 100,000 inhabi-tants)**	**(%)**
Total hospitalization risk effect	-3065	100	-3159	100	-2982	100
A00-B99 Certain infectious and parasitic diseases	-218	7.1	-218	6.9	-219	7.3
C00-C97 Malignant neoplasms	-141	4.6	-116	3.7	-170	5.7
D00-D48 In situ neoplasms, benign neo-plasms, neoplasms of uncertain or un-known behaviour, Diseases of the blood and haematopoietic organs and certain disorders involving the immune system	-71	2.3	-80	2.5	-63	2.1
E00-E90 Endocrine, nutritional and meta-bolic diseases	-95	3.1	-104	3.3	-86	2.9
F00-F99 Mental and behavioural disor-ders	-51	1.7	-44	1.4	-58	1.9
G00-G99 Diseases of the nervous system	-144	4.7	-132	4.2	-157	5.3
H00-H95 Diseases of the eye and ocular appendages and diseases of the ear and mastoid process	-115	3.8	-129	4.1	-101	3.4
I00-I99 Diseases of the circulatory sys-tem	-423	13.8	-416	13.2	-433	14.5
J00-J99 Diseases of the respiratory sys-tem	-314	10.2	-328	10.4	-302	10.1
K00-K93 Diseases of the digestive sys-tem	-337	11.0	-327	10.4	-348	11.7
L00-L99 Diseases of the skin and subcu-tis	-80	2.6	-72	2.3	-88	3.0
M00-M99 Diseases of the musculoskele-tal system and connective tissue	-370	12.1	-429	13.6	-310	10.4
N00-N99 Diseases of the urogenital sys-tem	-154	5.0	-175	5.5	-136	4.6
O00-O99 Pregnancy, childbirth and puer-perium	-50	1.6	-97	3.1	0	0.0
P00-P96 Certain states originating in the perinatal period	1	0.0	2	-0.1	1	0.0
Q00-Q99 Congenital malformations, de-formities and chromosomal anomalies	-12	0.4	-11	0.3	-13	0.4
R00-R99 Symptoms and abnormal clini-cal and laboratory findings not elsewhere classified	-206	6.7	-222	7.0	-190	6.4
S00-S99 Injuries	-210	6.9	-193	6.1	-226	7.6
T00-T98 Poisoning, and certain other consequences of external causes	-69	2.3	-64	2.0	-74	2.5
Z00-Z99 Factors influencing health status and leading to use of health services	-7	0.2	-5	0.2	-10	0.3
U00-U99 key for special purposes	1	0.0	1	0.0	1	0.0

The time periods 2005-2019, 2005-2014, 2014-2019, and 2019-2021 are shown for the total population and by sex – *ICD-10-GM* 10th revision of the International Classification of Diseases, German Modification
