# Supplementary material for: Population aging and changing hospitalization risks in Germany: a decomposition of changes in inpatient cases, 2005–2021
Source: BMC Public Health. 2026 Apr 30;26:1437. doi: 10.1186/s12889-026-27522-x (PMC13135272; doi:10.1186/s12889-026-27522-x)
Supplement: Supplementary file 5 — Figure comparing the development of hospital case numbers in Germany with the G-DRG analysis sample of the current study. [file 12889_2026_27522_MOESM5_ESM.pdf]

Development of hospital case numbers

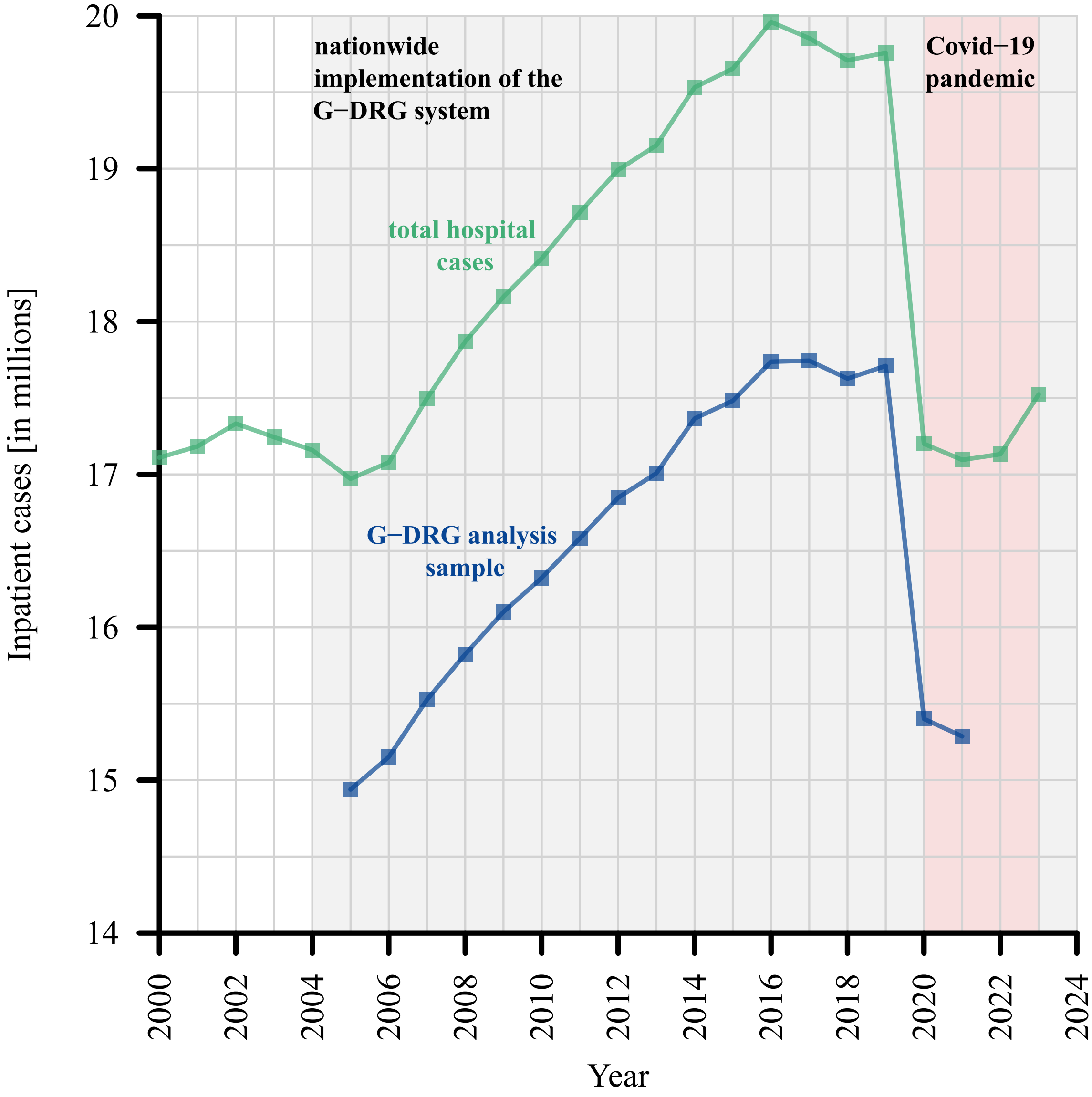

Fig. S3 Development of total hospital case numbers and inpatient case numbers from the G-DRG analysis sample – The total number of hospital cases was adjusted for patients with foreign or unknown residence based on statistic 23131-0005 ("Hospital Patients: Germany, Years, Gender, Residence of Patient") from the Federal Statistical Office, which can be found at: https://www-genesis.destatis.de/datenbank/online. The G-DRG analysis sample was adjusted for day cases, patients with foreign and unknown residence, missing values in age, sex, and cause of discharge, error DRG cases, and confidential cases, based on the G-DRG statistic (2005-2021)
